# Feasibility of a 20-gauge ProCore needle in EUS-guided subepithelial tumor sampling: a prospective multicenter study

**DOI:** 10.1186/s12876-018-0880-1

**Published:** 2018-10-19

**Authors:** Do Hoon Kim, Gwang Ha Kim, Chang Min Cho, Chang Hwan Park, Soo-Young Na, Tae Hyeon Kim, Yu Kyung Cho, Ji Hyun Kim, Dong-Wan Seo, Korean EUS Study Group

**Affiliations:** 1 Department of Gastroenterology, University of Ulsan College of Medicine, Asan Medical Center, 88 Olympic-ro 43-gil, Songpa-gu, Seoul, 05505 South Korea; 2 Department of Internal Medicine, Pusan National University School of Medicine and Biomedical Research Institute, Pusan National University Hospital, 179 Gudeok-ro, Seo-gu, Busan, 49241 South Korea; 3 Department of Internal Medicine, Kyungpook National University School of Medicine, 130 Dongdeok-ro, Jung-gu, Daegu, 41944 South Korea; 4 Department of Internal Medicine, Chonnam National University Medical school, 42 Jebong-ro, Donggu, Gwangju, 61469 South Korea; 5 Department of Internal Medicine, Jeju National University School of Medicine, 15, Aran 13-gil, Jeju-si, Jeju, Jeju-do 63241 South Korea; 6 Department of Gastroenterology, Wonkwang University School of Medicine, 895 Muwang-Ro, Iksan, Jeonlabuk-do, Iksan, 54538 South Korea; 7 Department of Gastroenterology, The Catholic University of Korea, Seoul St. Mary’s Hospital, 222 Banpo-daero, Seocho-gu, Seoul, 06591 South Korea; 8 Department of Gastroenterology, Inje University Pusan Paik Hospital, Bokji-ro 75, Busangjin-gu, Busan, 47392 South Korea

**Keywords:** Biopsy, Endoscopic ultrasonography, Gastrointestinal tract, Subepithelial tumor

## Abstract

**Background:**

Endoscopic ultrasonography-guided fine-needle biopsy (EUS-FNB) may facilitate tissue acquisition for a definitive diagnosis of gastrointestinal subepithelial tumors (SETs). This study aimed to determine the diagnostic yield of EUS-FNB using a novel 20-gauge ProCore needle with a coiled sheath in tissue sampling of gastrointestinal SETs.

**Methods:**

Between July 2016 and February 2017, 39 patients with gastrointestinal SETs were prospectively recruited from six university hospitals in Korea. Hypoechoic SETs ≥2 cm in size and originating from the submucosal and/or muscularis propria layer under EUS were eligible. This study was registered on ClinicalTrials.gov (NCT02884154).

**Results:**

A total of 36 patients were included in the final analyses. EUS-FNB was diagnostic in 88.9% of SETs. Tissue adequacy was judged as optimal in 97.2% of FNB specimens according to on-site visual evaluation by endosonographers, and in 88.9% of specimens according to pathologists. A macroscopically optimal core sample was obtained with two needle passes in 94.4% of cases. Technical failure rate was encountered in two cases (5.6%) after two needle passes. There were two cases (5.6%) of bleeding, which was managed endoscopically.

**Conclusions:**

EUS-FNB using a 20-gauge ProCore needle is a technically feasible and effective modality for histopathologic diagnosis of gastrointestinal SETs, providing adequate core samples with fewer needle passes;ClinicalTrials.gov number, NCT02884154.

## Background

Gastrointestinal subepithelial tumors (SETs) include benign, potentially malignant, and malignant lesions. Endoscopic ultrasonography (EUS) is a useful tool for the characterization of SETs, providing details on the gastrointestinal wall structures in addition to morphologic features [1]. However, the differential diagnosis of hypoechoic SETs remains challenging, and a definitive diagnosis can rarely be established on imaging modalities alone [2]. As the treatment decision for patients with SETs largely depends on the histopathological diagnosis, effective and high-quality tissue acquisition is required.

In this context, EUS-guided sampling methods, including fine-needle aspiration (FNA), Trucut biopsy (EUS-TCB), and fine-needle biopsy (EUS-FNB) have been introduced [3]. EUS-FNA with a 22-gauge or 25-gauge needle provides cytological aspirates, with a diagnostic yield of 70% to 79% [4, 5]. Alternatively, tissue cores can be procured with EUS-TCB, which enables immunohistochemical analysis and thus facilitates a definitive diagnosis [6]. However, the diagnostic yield of EUS-TCB is not higher than that of EUS-FNA, and technical failures frequently occur [5, 7]. The Trucut needle is relatively stiff, and the firing mechanism produced by the torqued echoendoscope limits its maneuverability and accessibility in certain locations within the gastrointestinal tract, such as the gastric antrum or duodenum [8].

Recently, a novel EchoTip ProCore high definition ultrasound biopsy needle with a coiled sheath has become available. This needle, which has a unique reverse bevel technology, allows for a simultaneous core biopsy to be obtained along with aspirated material. The 19-gauge ProCore needle was shown to obtain histologically adequate samples in nearly 90% of cases, with excellent technical feasibility in both intra-intestinal and extra-intestinal mass lesions [9]. However, as this 19-gauge needle is still stiff, its maneuverability is not satisfactory in the gastric antrum or duodenum. Furthermore, the diagnostic yield of the 22-gauge ProCore needle was found to be 81.8% to 86.0% in gastric SETs [10–12]. The novel 20-gauge needle offers improved flexibility for those more difficult EUS-FNA biopsy approaches, with easy to-and-fro passage of the needle, along with the benefits of the larger 20-gauge needle to yield histologic grade tissue.

Although there is evidence supporting the utility of these procedures, diagnostic yields have varied according to the location in the gastrointestinal tract and specific needle types. The aim of this prospective multicenter study was to investigate the feasibility and diagnostic yield of EUS-FNB with a 20-gauge ProCore biopsy needle in the diagnosis of gastrointestinal SETs.

## Methods

### Patients

Consecutive patients with gastrointestinal SETs were prospectively enrolled at six university hospitals in Korea (Asan Medical Center, Pusan National University Hospital, Kyungpook National University Hospital, Chonnam National University Hospital, Jeju National University Hospital, and Wonkwang University Hospital) from July 2016 to February 2017. Those with a hypoechoic SET ≥2 cm in size and located in the submucosa and/or muscularis propria layer under EUS examination were eligible. Exclusion criteria were as follows: (i) anechoic or hyperechoic SETs on EUS that suggested cyst, vessel, or lipoma; (ii) those with a history of coagulopathy, presenting as a platelet count < 50,000/mm^3^ or prothrombin time < 50%. All enrolled patients provided written informed consent for their participation in the study. The study protocol was approved by the institutional review board of each hospital and was conducted in accordance with the Declaration of Helsinki and its amendments, and the Good Clinical Practice guidelines. This study was also registered on ClinicalTrials.gov (NCT02884154). All authors had access to the study data and reviewed and approved the final manuscript.

### Procedure technique

All procedures were performed using a linear array echoendoscope (GF-UCT 140, UCT 240 or UCT 260; Olympus, Tokyo, Japan) with the patient under conscious sedation. After the target lesion was endosonographically visualized, a 20-gauge ProCore needle (EchoTip ProCore; Wilson-Cook Medical Inc., USA) was advanced into the target tissue. The key features for 20-gauge ProCore needle are (1) the core trap designed for receiving tissue into needle, (2) Menghini bevel for obtaining sample, (3) the coiled sheath to facilitate needle flexibility and (4) the ReCoil stylet aids stylet management minimizing the risk of contamination. After successful puncture, the endosonographer moved the needle to and fro within the lesion for more than 10 to 15 times, while an assistant simultaneously slowly pulled out the stylet over 30 to 60 s without suction to achieve minimal negative pressure within the needle.

At least three needle passes were performed using the designated needle, and the number of required needle passes to obtain sufficient core tissue was recorded. If such samples were not obtained after three needle passes, the number of required passes was considered to be four. Technical failure was defined as malfunction of the needle before three needle passes. Diagnostic failure was defined as failure to obtain sufficient core samples after three passes. If a diagnostic or technical failure was encountered, an alternative needle was used, according to the judgment of the endosonographers. In the rescue cohort, a maximum of three passes were attempted using the alternative needle, until either sufficient core samples were obtained or technical failure was encountered.

### Histological analysis

**Fig. 1:**
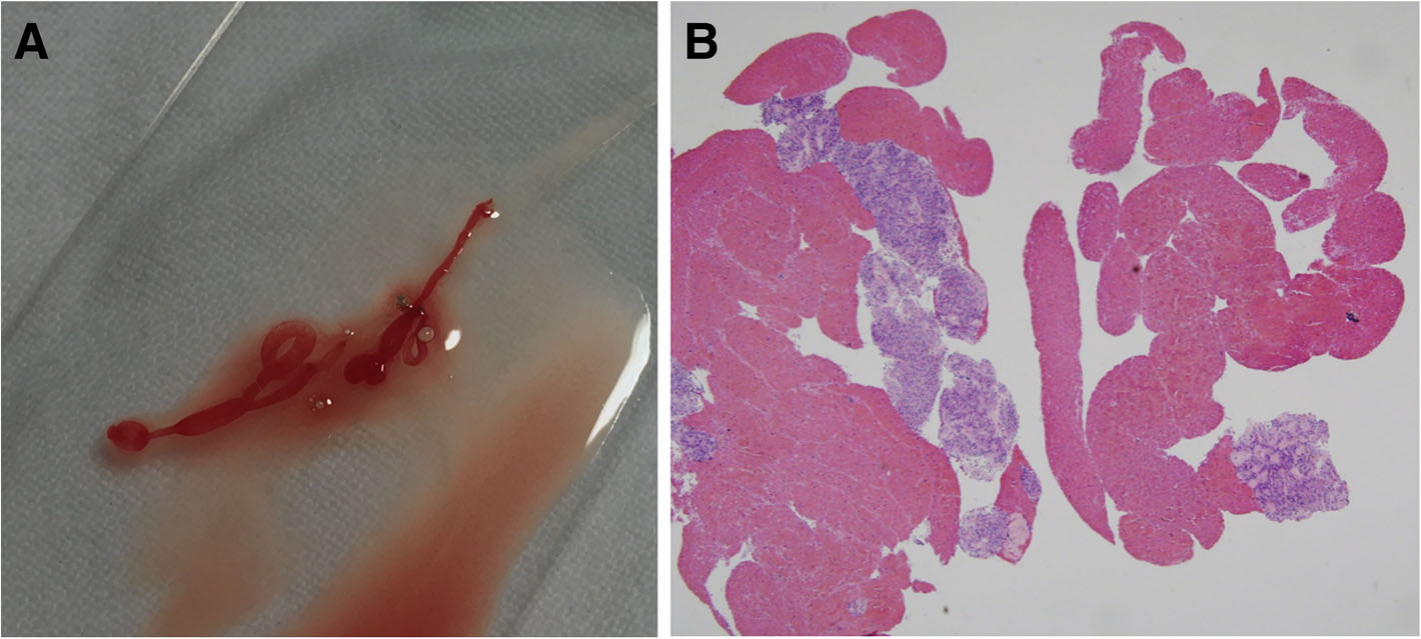
Macroscopic and microscopic specimens from endoscopic ultrasound-guided fine-needle biopsy with a 20-gauge ProCore needle. **a** Gross findings of the endoscopic ultrasonography-guided fine needle biopsy specimen. **b** Hematoxylin and eosin stain from the core biopsy of a gastrointestinal stromal tumor (× 40)

As pathologists were absent during endoscopy, biopsy samples were harvested and stored by the endosonographers for subsequent processing. The specimens were expelled onto glass slides by re-insertion of a stylet or by flushing air into the needle assembly. The endosonographers then carefully inspected the materials on the slides and determined whether tissue cores were present, with these being defined as whitish pieces of tissue with apparent bulk (Fig. 1a). The core samples were macroscopically assessed according to the following types: (i) definite tissue core, (ii) visible tissue core mixed with blood clots, or (iii) only blood or scant sample without any tissue core. The former two sample types were considered macroscopically optimal. If tissue cores were obtained, they were lifted off with a filter paper strip and placed in a formalin solution for hematoxylin and eosin staining. Samples with tissue cores were graded as optimal or suboptimal under pathological examination (Fig. 1b): optimal if the material allowed satisfactory assessment of histologic architecture and immunohistochemical evaluation, and suboptimal if the core sample was inadequate for the abovementioned assessments. After the core biopsy sample was harvested or if a core sample was unavailable, some aspirated samples were smeared and fixed in 95% absolute alcohol for cytological analysis including Papanicolaou staining. Cytological material was sent to the cytologists as a fixed or an air-dried slide.

A pathologic diagnosis was based on hematoxylin and eosin with or without immunohistochemical staining, such as CD34, CD117, S100, and smooth muscle actin. Because the morphological characteristics of mesenchymal tumors are nonspecific, a positive diagnosis by EUS-FNB was only considered true positive when immunohistochemical analysis was conclusive. In patients who underwent endoscopic or surgical resection of the tumors, the final diagnosis was based on histopathological assessment of the resected specimen. Otherwise, histopathological assessment of the FNB samples was deemed to be the gold standard.

### Outcome parameters

The primary objective was to determine diagnostic accuracy. Diagnostic accuracy was defined that the sufficient samples were obtained for satisfactory assessment of histologic architecture and immunohistochemical evaluation within three needle passes. Because the morphological characteristics of mesenchymal tumors are nonspecific, a positive diagnosis by EUS-FNB was only considered true positive when immunohistochemical analysis was conclusive. The secondary outcome measures were the tissue adequacy, the number of needle passes required to obtain a sufficient tissue core, and the rates of diagnostic failure, technical failure, and adverse events. Tissue adequacy was defined as macroscopically and histologically optimal core samples. Adverse events were defined as any deviation from the clinical course after EUS-guided sampling, such as excessive bleeding at the site of puncture or perforation.

### Statistical analysis

The required sample size was estimated to be a minimum of 32, based on a two-tailed 95% confidence interval with a width equal to 0.298 and a diagnostic accuracy of 80%. Assuming a dropout rate of 20%, the final required sample size was calculated to be 38 patients.

Descriptive statistics were used to document the characteristics of SETs and procedure-related outcomes. Categorical variables were presented as frequencies and proportions. Continuous variables were summarized as median values with range or means with standard deviation. All statistical analyses were performed using SPSS version 21.0 (SPSS Inc., Chicago, IL).

## Results

**Fig. 2:**
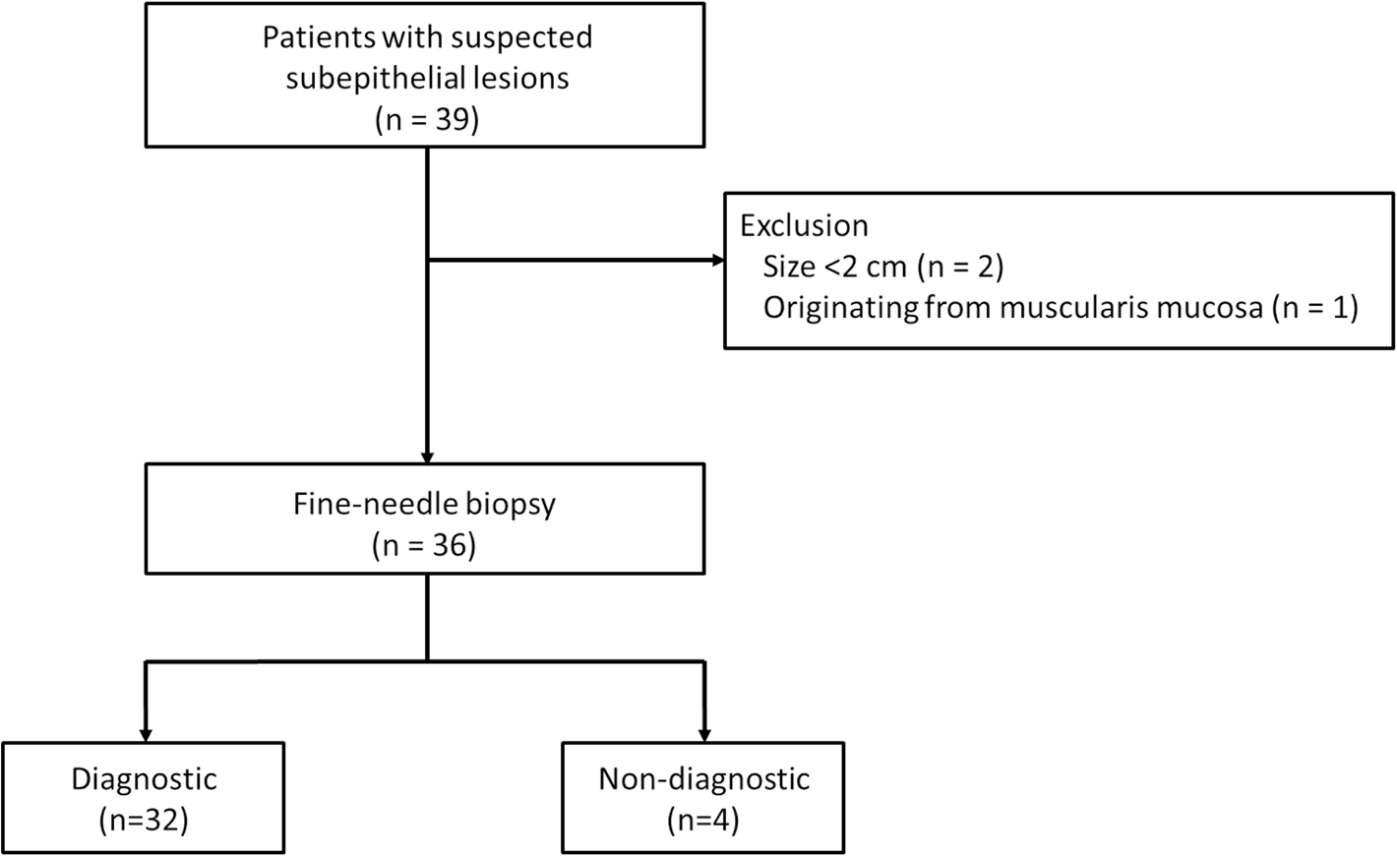
Flowchart of the study

Of the 39 enrolled patients, three were excluded from the final analyses: two had lesions that were suspected to be larger than 2 cm, but were found to be < 2 cm on EUS examination, and one lesion was revealed to originate from the muscularis mucosa layer. Ultimately, 36 patients were included in the analysis (Fig. 2). Demographic and clinical characteristics of the SETs are shown in Table 1.

**Table 1:** Baseline characteristics of the patients and subepithelial lesions

Age (years)	62.5 (27–82)
Male: female	19:17
Tumor size on EUS (cm)	2.5 (2.0–15.0)
Location	
Esophagus	4 (11.1)
Stomach	30 (83.3)
Cardia	3 (10.0)
Fundus	4 (13.3)
Body	18 (60.0)
Antrum	5 (16.7)
Duodenum	2 (5.6)
Originating layer	
Submucosa	6 (16.7)
Muscularis propria	27 (75.0)
Both layers	3 (8.3)
Final diagnosis	
Gastrointestinal stromal tumor	20 (55.6)
Leiomyoma	7 (19.4)
Heterotopic pancreas	2 (5.6)
Schwannoma	1 (2.8)
Glomus tumor	1 (2.8)
Carcinoma	2 (5.6)
No final diagnosis	3 (8.3)

Variables are presented as number (%) or median (range)

EUS, endoscopic ultrasonography

**Fig. 3:**
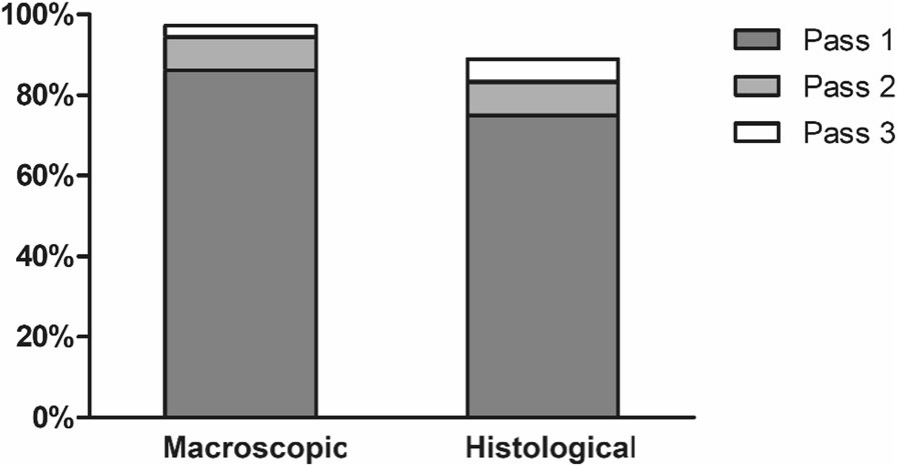
Tissue adequacy according to the needle passes

Procedural characteristics and outcomes are summarized in Table 2. The needle punctures were successful in all cases irrespective of tumor location. The EUS-FNB results were diagnostic accuracy in 88.9% (32/36) of SETs. Tissue adequacy was optimal in 97.2% (35/36) of FNB specimens according to the endosonographers’ on-site visual evaluations, and in 88.9% (32/36) of specimens according to the pathologists. The mean numbers of needle passes required to obtain optimal tissue core samples were 1.2 ± 0.6 macroscopically and 1.5 ± 1.0 histologically. The median number of needle passes was one for both evaluations. The tissue adequacy was macroscopically optimal in 86.1% of cases after the first needle pass, and each needle pass yielded adequacy rates over 85% (Table 3). The tissue adequacy increased as the number of needle passes increased, with macroscopically optimal core samples being obtained in 94.4% of patients after two needle passes (Fig. 3). Three cases were considered as optimal by the endosonographer but were revealed to be suboptimal on histopathological examination: there was insufficient submucosal tissue in two cases and only fibrotic tissue was found in one case.

**Table 2:** Procedural characteristics and outcomes of the EUS-guided fine-needle biopsies

Diagnosis achieved	32 (88.9)
Sample adequacy during three needle passes	
Macroscopic adequacy	35 (97.2)
Definite tissue core	32 (88.9)
Suspicious tissue core	3 (8.3)
Histological adequacy	32 (88.9)
Number of required needle passes to obtain optimal tissue core sample^a^	
Macroscopic	1.2 ± 0.6
Histological	1.5 ± 1.0
Technical failure	2 (5.6)
Adverse events	2 (5.6)

Variables are presented as number (%) or mean ± standard deviation

^a^If the optimal core sample was not obtained after three needle passes, the number of passes required was considered to be four

**Table 3:** Sample adequacy during three needle passes

	Pass 1 (*n* = 36)	Pass 2 (*n* = 36)	Pass 3 (*n* = 34)	Total (*n* = 36)
Macroscopic adequacy	31 (86.1%)	33 (91.7%)	32 (94.1%)	35 (97.2%)
Histological adequacy	27 (75.0%)	29 (80.6%)	27 (79.4%)	32 (88.9%)

Technical failure was encountered in two cases (5.6%) after two needle passes in the fundus and duodenal bulb. However, optimal tissue samples were obtained before the technical failure in both cases. In terms of adverse events, procedure-related bleeding occurred in two cases (5.6%). The bleeding was associated with needle puncture and was managed endoscopically with hemoclips.

Finally, 10 patients with diagnostic results avoided surgery: seven patients with leiomyoma, two with heterotopic pancreas, and one with glomus tumor. The diagnostic yield of EUS-FNB was 95.0% (19/20) in patients with a GIST. One lesion, which was not diagnosed on EUS-FNB, was surgically resected and confirmed as a GIST. Of 19 patients with a GIST, 13 had undergone surgical resection by the time of the analyses, and the histopathological diagnoses were consistent with the EUS-FNB diagnoses.

## Discussion

We investigated the diagnostic yield of EUS-FNB using a novel 20-gauge ProCore needle with a coiled sheath in the diagnosis of gastrointestinal SETs. Needle punctures were successful in all cases, irrespective of the location, and the diagnostic yield was 88.9%. The rates for obtaining macroscopically and histologically optimal core samples with EUS-FNB were 97.2% and 88.9%, respectively. The median number of needle passes for both macroscopic and histological adequacy was one. Our findings suggest that the 20-gauge ProCore needle may yield optimal core tissue samples with fewer needle passes compared with 22-gauge ProCore needle.

EUS-guided samplings are pivotal methods for tissue acquisition in gastrointestinal SETs, and a number of studies have evaluated the feasibility of EUS-guided sampling techniques in the diagnosis of SETs. EUS-FNA usually yields small sample volumes that are mainly processed for cytological evaluation [4, 5]. However, the cytological aspirate obtained by EUS-FNA is quantitatively low, and is often insufficient for differential diagnosis, especially in cases of gastric mesenchymal tumors that mandate an immunohistochemical assay. Samples with preserved tissue architecture are necessary to make a definitive diagnosis of hypoechoic SETs, especially when they are located in the muscularis propria layer. Although EUS-TCB provides large core tissue samples allowing histological examination as well as immunohistochemical staining, the Trucut needle is associated with technical difficulties because of its inherent stiffness, which results in a high technical failure rate [5, 6, 13]. Furthermore, the Trucut needle allows only one pass in a single axis, which thereby results in a limited diagnostic yield.

Recently, EUS-FNB technique has been developed, allowing core biopsy samples to be attained along with aspirated material. The ProCore needle is made of stainless steel, with a nitinol stylet and there is a reverse bevel to hook and cut tissue. Studies suggested that EUS-FNB may be advantageous for optimizing specimen adequacy and diagnostic accuracy. The 19-gauge ProCore needle showed histologic adequacy of 89.5% and diagnostic accuracy of 86.0% in the diagnosis of intra-intestinal and extra-intestinal mass lesions [9]. The diagnostic accuracy was 81.8–86.0% for gastric SETs when the 22-gauge needle was used [10–12]. In the present study, optimal macroscopic and histological core samples were procured in 97.2% and 88.9% of cases with three needle passes, which resulted in a high diagnostic histologic accuracy rate. Furthermore, adequate tissue core was obtained within two needle passes in most cases, with only 5.6% of cases requiring three needle passes to achieve a diagnosis.

The diagnostic yield of EUS-guided sampling depends on a variety of factors, such as the nature of the target lesion, site of the puncture, the availability of a cytopathologist, the experience of the endosonographer, and the type and size of the needle used [3]. Regarding the needle size, the large-caliber needles seem to have the advantage of acquiring more tissue, which enables the assessment of architectural features. However, a larger needle is prone to have technical difficulties with respect to its maneuverability and accessibility, whereas a smaller needle is flexible and can be fanned in multiple directions within the target lesion. Indeed, studies comparing the diagnostic performance for SETs did not demonstrate any significant advantage of EUS-TCB or EUS-FNB over a standard FNA needle in terms of cytologic parameters, amount of diagnostic cell block material, adequacy, and accuracy [4, 5, 14]. Of note, the number of needle passes required for diagnosis was significantly lower when using the ProCore needle, suggesting that a better quality sample was obtained in each pass [11, 14–16]. In the present study, the median number of needle passes to achieve both macroscopic and histological adequacy was one. The first pass of the 20-gauge ProCore needle yielded a histologically optimal tissue core in 75.0% of cases, and the histological tissue adequacy on each pass is over 75% throughout the procedures. These results demonstrate the high quality of the tissue obtained by a single pass of the ProCore needle.

It is sometimes difficult to determine whether sufficient core tissues are obtained during EUS-guided sampling, as indicated by the discrepancy between macroscopically and histologically assessed tissue adequacy. One possibility is that the visible materials do not consist of a tissue core, while another is that the materials acquired are not representative of the target lesion. The latter can be more challenging in clinical practice, because a tissue core may well be acquired but then revealed to give little information in respect to the diagnosis. In this present study, three cases were considered suboptimal for histological evaluation despite being macroscopically assessed as optimal core samples. All three cases contained visible tissue materials that seemed to be core samples, but were later revealed to be non-diagnostic. As most institutions do not have on-site pathologists, certain criteria for the macroscopic visual assessment of a specimen by the endosonographer can be helpful to ensure the adequacy of tissue cores and to reduce unnecessary punctures.

Although bleeding and perforation are potentially life-threatening adverse events of EUS and EUS-guided procedures, the incidence of adverse events has been reported as being low [17, 18]. In addition, most adverse events were caused by 19-gauge needles [7, 19]. Previous report using 22-gauge ProCore needles showed either no or low adverse event rates [10–12, 20]. In the present study, minor bleeding occurred during the procedure in two cases (5.6%), and was controlled endoscopically, thereby supporting the safety of EUS-FNB procedures.

Our study had several limitations of note. First, we included only academic centers with highly experienced endosonographers. Second, the size of all SETs included in our study was ≥2 cm. According to recent guidelines for SETs, when neoplastic SETs are 2–5 cm in diameter or when SET < 2 cm have clinically malignant features on endoscopy, the guidelines recommend detailed examination with EUS, computed tomography with contrast enhancement, and/or EUS-FNA. Clinically malignant features means irregular borders, ulceration, and/or growth during endoscopic follow-up. When there are no clinically malignant features, gastric SETs < 2 cm could be followed up by endoscopy or EUS once or twice a year until the tumors increase in size or become symptomatic, even if they are diagnosed as GISTs later on [21]. Therefore, we included only SETs with ≥2 cm in size. Third, in cases which do not need treatment surgically or endoscopically, the FNB results were determined as final diagnosis. There is a very rare possibility of malignant transformation in benign tumors, but it is a problem related to the natural history of tumors, not a wrong diagnosis. Fourth, cytological aspirates and histological core samples were not interpreted separately. In previous studies, the amount of diagnostic cell block material did not vary according to the use of either a beveled or standard needle, and the use of a beveled needle provided no benefit in terms of the diagnostic cell block [14]. Another possible limitation of our study is that the mitotic count and Ki-67 labeling index of the GIST were not determined. Although the diagnosis of GIST was successfully made before surgical resection, there may be considerable discrepancy in the mitotic count or Ki-67 index of the tumors between the EUS-FNB and surgical specimens [12].

## Conclusions

In conclusion, EUS-FNB using a 20G ProCore needle is a technically feasible, safe, and effective modality for histopathologic diagnosis of gastrointestinal SETs larger than 2 cm, providing adequate tissues core with fewer needle passes.

## Abbreviations

EUS: Endoscopic ultrasonography
EUS-FNB: Endoscopic ultrasonography-guided fine-needle biopsy
FNA: Fine-needle aspiration
GIST: Gastrointestinal stromal tumor
SET: Subepithelial tumor
TCB: Trucut biopsy
